# Importance of *N*-Acyl-Homoserine Lactone-Based Quorum Sensing and Quorum Quenching in Pathogen Control and Plant Growth Promotion

**DOI:** 10.3390/pathogens10121561

**Published:** 2021-11-30

**Authors:** Anton Hartmann, Sophia Klink, Michael Rothballer

**Affiliations:** 1 Host-Microbe-Interactions, Faculty of Biology, Ludwig-Maximilians-University München, Grosshaderner Str. 2–4, D-82152 Planegg-Martinsried, Germany; ahartmanndr@gmail.com; 2 Institute of Network Biology, Helmholtz Zentrum München-German Research Center for Environmental Health, Ingolstaedter Landstr. 1, D-85764 München-Neuherberg, Germany; sophia.klink@helmholtz-muenchen.de

**Keywords:** quorum sensing, quorum quenching, *N*-acyl-homoserine lactones, AI-2, biological control, plant perception, field application

## Abstract

The biological control of plant pathogens is linked to the composition and activity of the plant microbiome. Plant-associated microbiomes co-evolved with land plants, leading to plant holobionts with plant-beneficial microbes but also with plant pathogens. A diverse range of plant-beneficial microbes assists plants to reach their optimal development and growth under both abiotic and biotic stress conditions. Communication within the plant holobiont plays an important role, and besides plant hormonal interactions, quorum-sensing signalling of plant-associated microbes plays a central role. Quorum-sensing (QS) autoinducers, such as *N*-acyl-homoserine lactones (AHL) of Gram-negative bacteria, cause a pronounced interkingdom signalling effect on plants, provoking priming processes of pathogen defence and insect pest control. However, plant pathogenic bacteria also use QS signalling to optimise their virulence; these QS activities can be controlled by quorum quenching (QQ) and quorum-sensing inhibition (QSI) approaches by accompanying microbes and also by plants. Plant growth-promoting bacteria (PGPB) have also been shown to demonstrate QQ activity. In addition, some PGPB only harbour genes for AHL receptors, so-called *luxR*-solo genes, which can contribute to plant growth promotion and biological control. The presence of autoinducer solo receptors may reflect ongoing microevolution processes in microbe–plant interactions. Different aspects of QS systems in bacteria–plant interactions of plant-beneficial and pathogenic bacteria will be discussed, and practical applications of bacteria with AHL-producing or -quenching activity; QS signal molecules stimulating pathogen control and plant growth promotion will also be presented.

## 1. Introduction

During the evolution of land plants, plant-associated microbiomes co-evolved and were integrated into microbe–plant holobionts. The functions of these plant holobionts, including the plant and the associated microbiome, are based on gene expression of all partners. The constitution of the holobiont is dependent on specific signalling and perception events between the microbial and plant partners. The response of plants to abiotic and biotic stressors is positively modulated in association/symbiosis with plant-beneficial microbes, resulting in substantial plant growth promotion. Besides microbially produced plant hormones and a diversity of other metabolites, bacterial quorum-sensing (QS) signalling substances, e.g., of the *N*-acyl-homoserine lactone (AHL, AI-1) type, play a significant role in beneficial bacteria–plant interaction and pathogen defence [1]. During the course of bacteria–plant interactions, microbial signalling molecules stimulate the priming of specific plant gene expression, leading to enhanced pathogen defence and growth promotion [1].

The QS-based regulation of gene expression in bacterial populations is based on the density of bacterial populations and the surrounding environmental conditions. For more than 2 decades, it has been known that bacteria are social organisms, communicating with each other through diffusible small molecules [2,3]. A very sensitive and effective communication network within bacterial communities is based on autoinducer (AI) signalling. Signalling molecules of *N*-acyl-homoserine lactone (AHL or AI-1) type, hydrophilic (short CH-chain) and hydrophobic (long CH-chain), are frequent in Gram-negative bacteria and produced by the synthase LuxI [4,5,6]. Based on the specific perception of these autoinducers within cells by the LuxR-receptors, the density of not only their own population but also neighbouring populations with the same communication code is assessed. In addition, the quality of the environment is reflected in the AI concentration, and consequently, the expression of specific genetic potential of bacterial populations is optimized for these conditions. This makes autoinducer signalling an essential and evolution-relevant tool for the efficient control of gene expression and, thus, bacterial behaviour [7]. There is notable structural diversity among known QS autoinducers, for example, unusual AHL signals (such as *N*-carboxylated AHLs in the methanogenic archeon *Methanosaeta harundinacea*) [8], cinnamoyl HSL in *Bradyrhizobium* spp. [9], or p-coumaroyl HSL in *Rhodopseudomonas palustris* [10]. While AHL QS signals are mostly described in Gram-negative bacteria, borated tetrahydrofuranes, the so-called alternative autoinducer (AI-2), are found in a wide variety of Gram-negative and Gram-positive bacteria [11]. Other known QS signal molecules are butyrolactones in *Streptomyces* spp. [12] and 2-heptyl-3-hydroxy-4(1H)-quinolone (PQS) in *Pseudomonas* spp., regulating numerous virulence genes, including those involved in iron scavenging [13]. Some Gram-negative bacteria also produce diffusible signal factors (DSF), which are derived from fatty acids such as cis-2-unsaturated fatty acids (e.g., cis-11-methyl-2-dodecenoic acid of *Xanthomonas campestris pv. campestris*) [14]. DSF autoinducers also control the virulence of *Burkholderia cenocepacia* and *P. aeruginosa* [15]. Besides AI-2, Gram-positive bacteria mainly use small peptides as QS signals [2]. Furthermore, many bacteria are able to produce numerous volatile compounds, which can alter the growth and metabolism of bacteria and fungi, influence protist and arthropod behaviour, and impact plants and animals, as reviewed recently by Netzker et al. [16] As defined in 1904 by Lorenz Hiltner, the root environment (“rhizosphere”) is characterised by an ample supply of nutrients and is thus enriched by beneficial as well as pathogenic bacteria [17]. Many pathogenic bacteria (including plant pathogens) use QS autoinducing systems as major determinants of their virulence [18]. QS-regulated systems can be inhibited or quenched by different mechanisms, as reviewed by Uroz et al. [19] and, more recently, in detail by Grandclément et al. [20], indicating their great potential for biological control activity.

## 2. (Micro)evolution Mechanisms Affecting Bacteria-Plant Interactions and QS Signalling in Plant-Associated Bacteria

The co-evolution of microbes with emerging land plants constituted the functionality of efficient microbe–plant interactions. It has been demonstrated from genome sequences of the diverse and widely distributed rhizobacterial genus *Azospirillum* that a substantial amount of the genome has been collected from other bacteria that are efficient in plant colonisation [21]. It is only through these rhizosphere competence genes that *Azospirillum* could become a successful coloniser and be efficient in its interaction with plants. Since long ago, it has been a valid empirical approach to improve the rhizosphere competence of candidate inoculation strains by passage through the “rhizosphere school”. Horizontal gene transfer (HGT) in the phytosphere is known to occur frequently due to high metabolic activity, microbial density, and positive selection in the rhizosphere/phytosphere [22]. In addition, mobile genetic elements (MGE) such as plasmids of the Inc-P1-group can mediate the exchange between bacterial cells in the rhizosphere, and can thus contribute to the plasticity and dynamic of the genetic content of bacteria [23]. In addition, phenotypic switching (PS) is an ongoing process with much higher probability than HGT, plasmid transfer through MGE, or spontaneous mutations [24]. PS optimises niche adaptation in microbe–host interactions and is mostly known for pathogens, but it has also been found in plant-beneficial bacteria [25]. Phenotypic switches or variations effectively modify interactions of bacteria with higher organisms, specifically PGPB with plants. 

The plant-beneficial bacterium *Pseudomonas brassicacearum* underwent spontaneous phenotypic variation during the root colonisation of *Arabidopsis thaliana* and *Brassica napus in planta* and in vitro, resulting in different colony appearances on agar plates [25]. Phase II cells formed translucent colonies and could only be found at the surface of young roots and root tips, while wild-type cells (phase I cells) were located at the basal part of roots [26]. So-called phase II cells could spread faster on root surfaces due to the overproduction of flagellin; they also could swim and swarm, and thus, phenotypic variation is a strategy for gaining greater colonisation power. As regulatory elements controlling this phenotypic switching, frequent point mutations in the *gacS* or *gacA* gene were found [26]. Due to these point mutations in the *gacS-gacA* system, several genes of the secondary metabolism and the small RNA genes were differentially expressed, causing a downregulation of three different AHLs, biofilm formation, antifungal secondary metabolites, type VI secretion machinery, and alginate and indole acetate biosynthesis, as well as the exoenzymes lipase and protease. It is reasonable that AHL-downregulation resulted in a reduced formation of biofilms and biosynthesis of secondary metabolites. Interestingly, the synthesis of AHL as a key regulatory signal molecule is involved in this central regulatory phenomenon. In plant-beneficial bacteria, such as *Azospirillum* spp., phenotypic variations were also observed, as reviewed by Wisniewski et al. [27]. For example, variants with enhanced exopolysaccharide production appeared after starvation stress. However, as far as is known, these variants had no impact on the ecological fitness of the bacteria [28]. Modifications in the QS system, such as the novel interkingdom signalling based on *luxR*-solo genes, may have resulted from diverse genetic evolutionary processes in *Azospirillum* and other rhizosphere bacteria; see [29] and below. These evolutionary processes can be expected to be permanently ongoing in the metabolically very active and microbiologically highly diverse rhizosphere habitat.

## 3. Role of Quorum Quenching (QQ) and Quorum-Sensing Inhibition (QSI) in Biological Control of Quorum-Sensing Active Pathogens

Four major mechanisms of QQ are known, as summarized in Table 1. Degradation of the QS compounds is the most frequently found biological control activity [30]. It has been claimed that degradation of QS signals could effectively prevent the emergence of pathogens and cure rhizosphere soil [30]. In particular, members of the Gram-positive genus *Bacillus* spp. excrete high amounts of AHL lactonases, such as AiiA (autoinducer inactivation A), which can attenuate the virulence of the plant pathogen *Erwinia carotovora* [31]. Since *Bacillus* spp. and other Gram-positive bacteria, which do not produce AHLs, are inhibited by high levels of 3-oxo-C12-homoserine lactone (3-oxo-C12-HSL), the ecological role of AiiA in Gram-positive bacteria is probably the detoxification of high AHL concentrations [32]. Using a “biostimulation” approach with the AHL analogues caprolactone or gamma heptalactone, AHL-degrading *Rhodococcus erythropolis* populations are stimulated to efficiently colonise plant roots [33]. This type of lactonase-active bacteria turned out to be beneficial biocontrol organisms able to protect crops such as potato against the phytopathogen *Pectobacterium* [34]. Interestingly, the nitrogen-fixing symbiotic bacterium *Ensifer* sp. strain NGR234 harbours multiple loci for the quorum quenching of *N*-acyl homoserine lactones [35]. Concerning the AI-2 system, highly effective inhibition of biofilm formation has been described for a metagenome-derived AI-2-quenching enzyme [36]. In addition to lactonases and amidases/acylases [30], AHL reductases convert 3-oxo-AHLs to 3-hydroxy-AHLs [37]. Cytochrome oxidases performing the oxidation of the acyl chain [38] have been described in bacteria, archaea, and eukaryotes. In invertebrates and especially mammals, several types of lactonases (called paraoxonases) are known to have important functions in controlling QS-active human-pathogenic *P. aeruginosa* [39]. Some Gram-negative bacteria, such as *Agrobacterium* and *Pseudomonas*, which actively produce several types of AHL autoinducers, are known to degrade their own QS signals through lactonases or amidases [40,41]. It can be assumed that they control the accumulation and also timing of QS AHL signalling through the activation of degrading activitiy to further optimise their functions in the prokaryote–eukaryote interaction. More recently, AHL-hydrolysing activity was discovered in the plant growth-promoting rhizobacterium *P. putida* IsoF [42]. Using highly resolving metabolite and immunological analysis, the dynamics of C12-HSL and its degradation product C12-homoserin could be quantified [43]. Among *A. brasilense* strains, which are very frequently and successfully applied as plant growth-promoting bacteria in practical agriculture in South America [44], the production of AHL autoinducers is very scarce [45]. *A. brasilense* Az39 was recently demonstrated to be able to degrade C3-unsubstituted AHLs (C4 to C12) as well as AHLs with hydroxy (OH-) and keto (oxo-) substitutions [46]. It can be assumed that this AHL-degrading activity supports the competitiveness of Az39 in root colonisation and may contribute to controlling AHL-active plant pathogens. Thus, the interruption of the QS communication within the rhizosphere community including competing rhizobacteria and plant pathogens could support plant growth-stimulating activity through a plant growth-promoting bacterium such as *A. brasilense* Az39. 

In addition to the enzymatic degradation of the quorum-sensing signals, blocking signal synthesis is another potent way to inhibit QS. The AHL-based QS system TofI/TofR in the rice pathogen *Burkholderia glumae* is inhibited by the TofI-inhibitor J8-C8. This is a synthetic analogue of C8-HSL and, therefore, a competitive inhibitor of toxoflavin production [47]. The biosynthesis of AI-2 is inhibited by the natural product fimbrolide and several alkyl-DPD analogues, leading to a reduction in biofilm formation of *Salmonella typhimurium* [48]. Farnesol, a metabolite of the fungus *Candida albicans*, leads to an inhibition of PQS and pyocyanin biosyntheses in *P. aeruginosa* [49]. 

A major strategy for avoiding or reducing QS-regulated bacterial virulence is the blockage of the receptor by structural analogues of the QS-signalling molecule. For example, different metabolites have been identified in edible plants and fruits inhibiting the *N*-acyl-homoserine lactone QS system of *Chromobacterium violaceum* and *P. aeruginosa* [51]. Flavonoids have been found to suppress the virulence of *Pseudomonas aeruginosa* through the allosteric inhibition of the LuxR-receptor [52]. Dihydropyrrolones, synthesised via the lactone-lactam conversion of lactone intermediates, were active as inhibitors of QS by inhibiting biofilm formation and the growth of *P. aeruginosa* [53]. State-of-the-art and future perspectives for anti-quorum-sensing agents of small molecules for inhibition also towards AI-2 QS signalling were reviewed by Guo et al. [55]. Furthermore, ambuic acid, a secondary metabolite of fungi, inhibited the biosynthesis of cyclic peptid quormones and gelatinase biosynthesis activity in *Enterococuus faecalis* and, e.g., the virulence activation in pathogenic *Staphylococcus aureus* and *Listeria innocua* [50]. Singh et al. [56] suggested a rationale to design structures of quorum-quenching peptides targeting the two component virulence (*VirSR)* system of *Clostridium perfringens.* This could lead to antipathogenic drugs targeting QS-mediated virulence expression. Finally, QS signalling can also be inhibited via signal transport by binding AHL-directed antibodies to the QS-signalling molecule [54]. The 3-oxo-C12-HSL-mediated QS signalling of *P. aeruginosa* was blocked by these antibodies. Several approaches have also led to functional AHL-targeting antibodies directed towards C10-HSL [57]. 

Many studies in the QQ and QSI areas have been directed at blocking the QS system, especially that of human-pathogenic bacteria. The appearance of multiple resistances against conventional antibiotic therapy has become a major health threat. Since quorum sensing is of central importance for virulence development of many pathogenic bacteria, the different modes of QS inhibition could be a potential strategy for inhibiting these pathogens in their interaction with the host without selecting resistances because general growth abilities of pathogens are not affected. The biocontrol of plant-pathogenic bacteria should benefit from these advances in the medical field (and vice versa).

## 4. Role of Quorum-Sensing Autoinducer *N*-Acyl-Homoserine Lactones and AHL-Producing Rhizobacteria Stimulating Biological Control and Plant Growth Promotion

AHLs are frequently used by both pathogenic and beneficial rhizobacteria to either optimise their virulence or their beneficial activity. This is, e.g., reflected by the finding that LuxI/R-QS circuits were found to be prevalent in microbiomes of *Populus* [58]. Plants should be able to detect and respond to these signalling compounds in a very early phase of interaction. Table 2 summarizes mechanisms of beneficial plant responses towards AHL-quorum sensing. The first evidence for specific plant responses to *N*-acyl homoserine lactones was found in the leguminous plant *Medicago truncatula* by Mathesius et al. [59]. The effects of 3-oxo-C14-HSL from symbiotic *Ensifer meliloti* and 3-oxo-C16:1-HSL from *P. aeruginosa* on the gene expression of *M. truncatula* were studied with a proteomic approach. The AHLs induced auxin-responsive and flavonoid synthesis proteins. They also stimulated the secretion of small compounds mimicking QS signals, and thus, may disrupt QS in associated bacteria [59]. In tomato plants, inoculated with the C6- and C8-HSL-producing *Serratia liquefaciens* MG1 and *P. putida* IsoF, pathogen defence and biological control activities towards the fungal pathogen *Alternaria alternata* were identified [60]. In plants inoculated with wild-type MG1 and IsoF cells, salicyclic acid was increased in the leaves and the SA- and ethylene-dependent defence genes (PR1a and chitinase) were induced; after inoculation with AHL-defective *S. liquefaciens* mutants, no similar effects were detected. In addition, it could be demonstrated that C6- and C8-HSL alone were able to induce disease suppression under axenic conditions [60]. Thus, also in this system, AHL-substances are sufficient to prime and induce plant defence activity in tomato plants. Applying fluorescently labelled *S. liquefaciens* MG1-producing AHLs in combination with AHL-reporter bacteria, the in situ production and distribution of AHLs could be monitored in detail [61]. The pathogen-antagonistic rhizosphere bacterium *S. plymuthica* HRO-48 could control the *Verticillium dahlia*-mediated wilt in oilseed rape (*Brassica napus*) [62]. The biosynthesis of C4-, C6-, and 3-oxo-C6-HSLs induced the production of extracellular chitinase and antifungal volatiles, which was abolished in lactonase-expressing transconjugants of *S. plymuthica* [62]. In *A. thaliana*, hydrophilic short-CH chain HSLs caused changes in the morphology of roots (e.g., increased root length) and changes in the phytohormone balance of roots in *A. thaliana* and barley [1,63,64]. In a thoroughly performed system of plant stimulation experiments, Shrestha et al. showed that combinations with lipophilic long-CH chain HSL (e.g., C14- or C12-HSL) had the most pronounced biological control effect in *A. thaliana* against the plant pathogen *P. syringae pv. tomato* (Pst) [65]. Thus, these variable plant responses revealed that the plant perception was dependent on the plant species and the molecular structure of the AHL autoinducer.

In *A. thaliana*, GCR1/GPA1 genes, which are involved in heterotrimeric G-protein mediated cell signalling of extracellular environmental signals to cells, were identified as the genetic basis for the root growth stimulation by short-CH chain C6- and C8-AHLs [66]. Mutants in GCR1 were insensitive to root length stimulation by C6- and C8-HSL, while overexpression resulted in increased root growth effects. In several experimental approaches, it has been demonstrated that water-soluble AHLs are taken up by plants through the plant vascular system into the shoot during a cellular energy consuming process [67,68]. AHL uptake was only found in plants such as *A. thaliana*, wheat, or barley, which are devoid of AHL-degrading enzymes, such as lactonases. In plant shoots, hydrophilic AHLs are able to modify the activity of several enzymes, including anti-oxidative capacity and xenobiotic phase II detoxifying enzymes improving stress tolerance [68]. Although hydrophobic AHLs (such as 3-oxo-C14-HSL) are not taken up by plants, they confer resistance towards the obligate biotrophic fungus *Golovinomyces orontii* in *A. thaliana* and *Blumeria graminis* in barley as well as towards the hemibiotrophic bacterial pathogen *P. syringae pv. tomato* DC3000 [69]. It was shown that in the presence of the bacterial elicitor flg22, the activation of the mitogen-activating protein kinases AtMPK3 and 6 was greater and followed by an enhanced expression of WRKY22 and 29 plus PR1a. Most interestingly, AtMPK6 was required for AHL-induced resistance, since deletion mutants failed to show the induced resistance phenotype [69]. The AHL-dependent priming of anti-oxidant gene expression and plant defence activity by hydrophobic AHLs with long CH chains (e.g., 3-oxo-C12-HSL or 3-oxo-C14-HSL) occurred via the oxylipin and salicylic acid signalling pathway [70]. Priming of defence responses was also shown to be a strategy to enhance resistance towards leaf rust in barley [71]. Interestingly, genetic differences were revealed in a range of barley lines, determining the responsiveness to 3-oxo-C14-HSL in the priming process for resistance against powdery mildew [72]. Most recently, a specific membrane-located receptor for the first interaction step with hydrophobic long-CH chain AHL was identified in *Arabidopsis thaliana* [77]. Moreover, the role of AHLs in pest insect control was very recently demonstrated by inoculation of two barley lines with 3-oxo-C14-HSL-producing *Ensifer meliloti* [73]. Inoculation with wild-type *E. meliloti* primed a reduced feeding and reproduction response of *Rhopalosiphum padi* aphids in comparison to AHL-negative mutant and control treatments [73]. An AHL-mediated suppression of sap-sucking insects in turn diminishes the transmission of plant viruses and thus contributes to plant health. The first successful field trials with wheat and the application of C6-HSL showed growth stimulation, demonstrating the possibility of practical applications for improving crop performance [78]. In addition, C4-HSL applied with carbon nanofibers induced enhanced growth, stress tolerance, and resistance against the fungal pathogen *Fusarium oxysporum* in chickpeas (*Cicer arientinum*) [79].

The AHL dependence of plant-beneficial and biological control effects have been demonstrated in several PGPB mutants lacking the *luxI*- or *luxR*-gene and in AHL-depleted transconjugants. Plant growth-promoting *Rhizobium radiobacter* F4 was first described as an endofungal bacterium of the plant growth-promoting fungus *Serendipita indica* (syn. *Piriformospora indica*) [75,80]. Even without the fungus, this bacterium proved to have similar plant-beneficial effects, e.g., stimulating abiotic and biotic stress tolerance [74]. Recently, it was demonstrated that *R. radiobacter* F4 produces a spectrum of short- and medium-CH chain AHL molecules [76]. Transconjugants with a lactonase-producing plasmid, devoid of AHLs, showed strongly reduced root colonisation in *A. thaliana* and barley, including only marginal plant-beneficial effects [76]. In the PGPB *Acidovorax radicis* N35 [81,82], producing OH-C10-HSL, *luxI*-deleted mutants were less effective in root colonisation. They also showed significantly different interaction with barley plants. While the AHL-producing wild type induced a plant expression profile of stimulation and some priming, the AHL-deleted mutant caused increased expression of defence responses, such as flavonoid biosynthesis [82]. Thus, in *A. radicis*, the AHL autoinducer may have an important role in influencing the perception of the host plant towards *A. radicis* N35 as a beneficial bacterium not to be inhibited. 

The beneficial influence of AHL autoinducers on plants was even present in bacteria without AHL-biosynthesis, as in most *A. brasilense* strains [46]. For example, in several successfully applied *A. brasilense* strains, such as Sp7, Sp245, Az39 or Ab-V5 and other *Azospirillum* strains, no AHL-producing *luxI*-genes were found, but *luxR*-solo or *luxR*-orphan genes were [45]. It was shown by Fukami et al. that the addition of 3-oxo-C6-HSL to *A. brasilense* Ab-V5 inoculants caused significant stimulation of plant growth promotion effects, while the degradation of added AHLs by lactonase blocked this stimulation [83]. Thus, external AHLs could stimulate plant responses through *luxR*-solo genes in *A. brasilense* Ab-V5. Since several *luxR*-solos could be detected in some of these strains, it was suggested that they may have evolved to perceive different metabolites of microbial or plant origin [84]. However, most of these plant or microbial signals, activating *luxR*-solos and the concomitant effect on bacteria–plant interaction, still need to be identified. For example, a *luxR* homologue in a cottonwood tree endophyte could activate gene expression induced by a plant signal or specific peptides [85]. The evolution of multiple *luxR*-solo genes may have resulted in receptors for specific interactions with metabolite signals from the plant to foster beneficial bacteria-plant interactions. 

## 5. Summary and Outlook

Recent findings related to AHL-based quorum-quenching activities and the presence of multiple *luxR*-solo genes [29] in plant growth-promoting bacteria have opened new insights into modified QS system activities with plant growth-promoting and biocontrol potential. However, many details are still unknown about the signalling metabolites of the accompanying rhizosphere microbiota and/or the plant host. Nevertheless, AHL compounds and the AHL-signalling plant-beneficial bacteria should be acknowledged as important elements in bacteria–plant interactions, leading to plant growth promotion and biological control of plant pathogens. Furthermore, the application of synthetic consortia of AHL-producing PGPBs supporting each other in the interaction with the host plant should be considered, when mixed inocula are constructed. This may extend to bacteria living endophytically. Recent publications have also highlighted that the application of *N*-acyl-homoserine lactones to plants for priming plant-beneficial activity could be a practical solution for improving plant performance in the field [78,79]. QS inhibitors applicable for quorum quenching are also very actively being developed against human-pathogenic bacteria as alternatives to conventional biocidal antibiotics. This should also lead to new application possibilities in agriculture, and in turn, QSI-based biocontrol of human pathogens should benefit from these experiences. Since rhizosphere and plant microbiomes, as well as their various associations, are very complex systems with multiple synergistic or inhibitory interactions, system biology approaches should be applied for further in-depth understanding [86]. In order to stimulate or inhibit specific quorum-sensing regulating circuits, synthetic biology tools should also be increasingly employed [87]. 

## Figures and Tables

**Table 1:** Overview of Quorum Quenching (QQ) and Quorum-Sensing Inhibition (QSI) activity.

(a) *N*-Acyl-Homoserine Lactone (AHL) Hydrolase/Oxido-Reductase Activity:			
Enzyme	Product(s)		References
AHL-lactonase	*N*-acyl-homoserine (HS)		[31,32,34,35,37,40,42,43,46]
AHL-acylase, amidase	Homoserine lactone plus fatty acid		[29,40,41]
AHL-oxido-reductase	Hydroxy-*N*-acyl-homoserine lactone		[38]
**(b) Quorum-sensing inhibitors:**			
**Inhibiting substance**	**Inhibiting mechanism**	**Target pathogen**	**References**
**Inhibition of signal synthesis:**			
TofI-Inhibitor J8C8	Inhibition of toxoflavin synthesis	*B. glumae*	[47]
Fimbrolide	Inhibition of AI-2 synthesis	*S. typhimurium*	[48]
Farnesol	Inhibition of PQS and pyocyanin synthesis	*P. aeruginosa*	[49]
Ambuic acid	Inhibition of cyclic peptide quormones	*S. aureus*	[50]
**Inhibition of autoinducer receptor:**			
Metabolites of plants /fruits	Inhibition of the AHL QS system	*P. aeruginosa*	[51]
Flavonoids	Allosteric inhibition of QS receptor	*P. aeruginosa*	[52]
Dihydropyrrolones	Inhibition of QS activity	*P. aeruginosa*	[53]
**Inhibition of signal transport:**			
AHL antibodies	Binding to AHLs and blockage of transport	*P. aeruginosa*	[54]

**Table 2:** Mechanisms of plant responses towards AHLs and AHL-producing bacteria.

AHL/AHL-Producing Bacterium	Plant Partner	Major Effects	Ref.
3-oxo-C12-HSL and 3-oxo-C16:1-HSL	*Medicago truncatula*	Induction of auxin-responsive and flavonoid synthesis proteins, secretion of QS-mimicking metabolites	[59]
*S. liquefaciens* MG1	Tomato	Induction of resistance against *Alternaria alternata*	[60]
*P. putida IsoF*			
C4-and C6-HSLs	Tomato	Increase in salicylic acid content, induction of PR1a and chitinase	[60]
*S. plymuthica* HRO-48, producing C4-, C6-, and oxo-C6-HSLs	Oilseed rape	Disease suppression of *Verticillium dahliae,* induction of antifungal volatiles and hydrolytic enzymes	[62]
C6- and C8-HSL	*A. thaliana*	Root growth stimulation through GCR1/GPA1 genes	[66]
C6- and C8-HSLs	*A. thaliana*	Transport into the shoot, change of phytohormone balance, multiple gene expression changes	[63]
C8- and C10-HSLs	Barley, wheat	Transport to the shoot, root changes, stimulation of anti-oxidative and phase II detoxifying enzymes	[64,67,68]
C12- and C14-HSLs	*A. thaliana*	Stimulation of expression of AtMPK3 and 6, WRKY22 and PR1a in the presence of the elicitor flg22	[69]
3-oxo-C14-HSL	*A. thaliana*	Priming of anti-oxidant and plant defence genes via oxylipin and salicylic acid pathway	[70]
3-oxo-C14-HSL	Barley	Priming of resistance towards leaf rust	[71]
3-oxo-C14-HSL	Barley	Enhanced resistance against powdery mildew, priming is genotype dependent	[72]
*E. meliloti*, producing 3-oxo-C14-HSL	Barley	Priming of reduced aphid feeding and reproduction	[73]
*R. radiobacter* F4, producing C8-, C10-, and C12-HSL (3-oxo- and OH-)	Wheat, *A. thaliana*	Strong induction of systemic resistance, induction of jasmonic acid and ethylene response	[74,75,76]

## Data Availability

Not applicable.
